# The relationship between secondary school students' English learning beliefs and English autonomous learning ability: a moderated mediation model

**DOI:** 10.3389/fpsyg.2026.1922753

**Published:** 2026-09-03

**Authors:** Bin Ying, Xiao Yu, Mingchen Wei, Yanling Liu, Xu Wang, Yibo Geng, Shuai Chen

**Affiliations:** 1 High School Affiliated to Southwest University, Chongqing, China; 2 Yuwang School, Bengbu, Anhui, China; 3 Faculty of Education, East China Normal University, Shanghai, China; 4 Southwest University Faculty of Psychology, Chongqing, China; 5 South China Normal University School of Psychology, Guangzhou, China

**Keywords:** English autonomous learning ability, English classroom enjoyment, English learning beliefs, English learning self-efficacy, secondary school students

## Abstract

**Introduction:**

This cross-sectional study examined the relationships among English learning beliefs (ELBs), English learning self-efficacy (ELSE), English classroom enjoyment (ECE), and English autonomous learning ability (EALA) among secondary school students.

**Methods:**

A convenience sample of 1,939 secondary school students was recruited from 11 provincial-level regions in China. Mediation and moderated mediation analyses were conducted using Hayes' PROCESS macro (Models 4 and 8) with 5,000 bootstrap samples, controlling for age, school stage, family residence, only-child status, and monthly family income.

**Results:**

After controlling for covariates, ELBs were positively associated with EALA (*B* = 0.731, 95% CI [0.701, 0.761]). A significant indirect association between ELBs and EALA via ELSE was observed (indirect association = 0.377; 95% bootstrap CI: [0.340, 0.414]). ECE significantly moderated the association between ELBs and ELSE (*B* = 0.036, 95% CI [0.017, 0.054]), such that the positive association was stronger at higher ECE levels. The index of moderated mediation was significant (index = 0.016, 95% bootstrap CI [0.007, 0.024]), indicating that the indirect association between ELBs and EALA through ELSE was stronger at higher levels of ECE.

**Discussion:**

These findings highlight the relevance of learning beliefs, self-efficacy, and classroom enjoyment to secondary school students' autonomous English learning.

## Introduction

In the current era of rapid educational reform and technological advancement, autonomous learning ability has become an important competence for secondary school students, enabling them to engage in lifelong learning across diverse contexts (Lee and Kim, 2020). The secondary school years represent an important period for the development of students' cognitive, emotional, and social capacities and for the formation of autonomous learning practices (Brown, 2002; Wang et al., 2024).

English, as a global lingua franca, occupies an important position in China's basic education system. Accordingly, developing students' English proficiency has long been an important educational goal in China. In recent years, basic education reform in China has increasingly emphasized educational quality and the cultivation of students' core competencies, including their autonomous learning ability. In 2020, the Ministry of Education issued the “General High School English Curriculum Standards (2017 Edition, 2020 Revision),” which identified four core competencies for English education: language ability, cultural awareness, thinking capacity, and learning ability. In these curriculum standards, “learning ability” refers to students' awareness and capacity to actively employ and adjust English learning strategies, make effective use of learning resources, and improve learning efficiency (Ministry of Education of the People's Republic of China, 2020). The English Curriculum Standards for Compulsory Education (2022 Edition) similarly emphasize students' ability to select and adjust learning strategies, use learning resources effectively, and develop appropriate learning habits (Ministry of Education of the People's Republic of China, 2022). These requirements highlight the importance of developing students' English autonomous learning ability (EALA).

In 2021, the General Office of the Central Committee of the Communist Party of China and the General Office of the State Council introduced the “Double Reduction” Policy, which aims to reduce excessive homework and off-campus tutoring for students in compulsory education. Within this policy context, greater emphasis has been placed on students' capacity to regulate and manage their own learning. Students' autonomous English learning may therefore be associated not only with external learning conditions but also with their internal psychological characteristics. In particular, how students understand English learning, how capable they perceive themselves to be in learning English, and how they experience the English classroom emotionally may all be closely associated with autonomous learning (Choi and Park, 2013; Shang and Kou, 2015). These psychological characteristics correspond, respectively, to English learning beliefs (ELBs), English learning self-efficacy (ELSE), and English classroom enjoyment (ECE).

Therefore, against the background of current curriculum reform and the Double Reduction Policy, the present study examines how these cognitive, motivational, and affective factors are associated with secondary school students' EALA.

## The present study

Understanding the factors associated with EALA among secondary school students has been a focal point of scholarly inquiry. Previous research suggests that EALA is associated with both learner characteristics and the social environment (Benson, 2013; Wen and Johnson, 1997). Learner characteristics can be categorized into intellectual and non-intellectual factors. Intellectual factors encompass perception and memory, while non-intellectual factors encompass learning beliefs, self-efficacy, and academic emotions (Shang and Kou, 2015). Because non-intellectual factors have been considered relatively malleable (Choi and Park, 2013), the present study focuses on ELBs, ELSE, and ECE as psychological factors associated with EALA. Recent studies involving secondary school language learners have also shown that autonomous and self-regulated learning are closely associated with cognitive, motivational, and emotional factors rather than merely reflecting students' use of learning strategies (Suwannaphim and Vibulphol, 2023; Wang et al., 2024).

### English learning beliefs and English autonomous learning ability

The study of English learning beliefs (ELBs) originated from a broader examination of language learning beliefs. According to Horwitz (1988), language learning beliefs encompass learners' cognitions, perspectives, attitudes, understandings, and assumptions regarding the nature and process of language learning. ELBs include beliefs about English learning aptitude, learning difficulty, the nature of English learning, learning and communication strategies, and learning motivation and expectations. According to self-regulation theory (Zimmerman, 2000), cognitive beliefs provide a foundational framework for understanding how individuals interpret learning tasks, select learning strategies, and monitor their learning processes. Learners with more adaptive beliefs (e.g., viewing language ability as malleable rather than fixed and recognizing communication as a central aspect of language learning) tend to report greater use of proactive self-regulatory strategies and higher levels of autonomous learning (Wen and Johnson, 1997).

Recent empirical research suggests that language learners' belief systems continue to evolve alongside modern learning environments, including AI-mediated and digital learning spaces, and remain closely associated with their self-directed learning behaviors (Wang et al., 2025). Shirzad et al. (2022), in a study of 300 Iranian high school students, found meaningful relationships among epistemic beliefs, self-efficacy, and language-learning strategies, further illustrating the relevance of learners' beliefs to adolescents' approaches to language learning.

Research in China has indicated that ELBs are closely associated with students' EALA, and that understanding students' learning beliefs may be important for understanding individual differences in autonomous learning (Chen and Guo, 2013). However, much of the existing research has focused on university students, whereas relatively few studies have examined secondary school English learners. Given that secondary school represents an important developmental period during which students' learning cognitions and approaches continue to develop, we propose:

Hypothesis 1: There is a positive association between secondary school students' ELBs and their EALA.

### The mediating role of English learning self-efficacy

English learning self-efficacy (ELSE) reflects a learner's subjective judgments of their capability to organize and execute actions required to achieve specific English learning goals (Bandura, 1993; Zhang and Yu, 2010). According to social cognitive theory (Bandura, 1997), ELBs may provide an important cognitive basis for students' self-efficacy judgments. Adaptive learning beliefs provide students with cognitive frames for evaluating their own capabilities. When students hold positive beliefs about the language learning process, they are more likely to report stronger perceptions of their capability to perform English learning tasks (Mo, 2020; Wei, 2021).

Furthermore, higher levels of English learning self-efficacy are likely to be associated with higher levels of English autonomous learning ability (EALA). According to self-efficacy theory, learners with stronger self-efficacy are more willing to set appropriately challenging learning goals, invest greater effort, persist when encountering difficulties, and actively select and adjust appropriate learning strategies (Bandura, 1993, 1997). These behaviors are highly consistent with the core components of autonomous learning, including goal setting, learning planning, strategy use, self-monitoring, and self-reflection (Zimmerman, 2000). Previous research has also found that English learning self-efficacy is significantly associated with learners' autonomous learning ability and related self-regulatory behaviors (Shang and Kou, 2015; Zhen et al., 2017).

Recent research has provided further support for the importance of self-efficacy in English learning among secondary school students. Li et al. (2024) reported that a self-efficacy-based intervention was associated with increases in students' self-efficacy, intrinsic motivation, and English proficiency, while Shirzad et al. (2022) found that self-efficacy was associated with both epistemic beliefs and language-learning strategies. These findings suggest that self-efficacy is not only an important individual psychological factor in English learning but may also statistically account for part of the association between learners' cognitive beliefs and their learning behaviors.

Taken together, ELSE may statistically mediate the association between ELBs and EALA. Accordingly, we propose:

Hypothesis 2: There are significant positive associations among ELBs, ELSE, and EALA, and ELSE statistically mediates the association between ELBs and EALA.

### The moderating role of English classroom enjoyment

With the introduction of positive psychology into the field of second language acquisition, increasing attention has been paid to positive emotions in foreign language classrooms. Among these emotions, English classroom enjoyment (ECE) has been recognized as an important positive academic emotion associated with learners' cognition, motivation, and learning behaviors (Dewaele and MacIntyre, 2016; Jin and Zhang, 2021). Unlike English learning beliefs (ELBs) and English learning self-efficacy (ELSE), which primarily reflect learners' internal cognitions and self-evaluations, ECE is more context-sensitive and is closely related to classroom activities, teacher support, peer interactions, and the overall classroom climate (Dewaele and Alfawzan, 2018).

According to the Broaden-and-Build Theory (Fredrickson, 2000, 2001), positive emotions broaden individuals‘ momentary thought-action repertoires and are associated with the deployment and accumulation of cognitive and psychological resources. In conjunction with the Affective Filter Hypothesis (Krahnke, 1983), an enjoyable classroom environment may coincide with reduced affective barriers, serving as a contextual boundary condition. When students report higher levels of ECE, the positive association between adaptive ELBs and self-efficacy judgments (ELSE) may be stronger (Villavicencio and Bernardo, 2016). In contrast, under conditions of low ECE, the positive association between ELBs and ELSE may be weaker. Previous research has likewise indicated that positive academic emotions are closely associated with learners' ELSE, learning engagement, and academic achievement (Ainley et al., 2005; Li and Wei, 2023; Villavicencio and Bernardo, 2016).

Accordingly, we conceptualize ECE as a moderator and propose the following hypothesis:

Hypothesis 3: ECE moderates the positive associations between ELBs and EALA, as well as between ELBs and ELSE.

### Study contribution

In sum, with a focus on non-intellectual factors associated with EALA, the present study examines the associations of secondary school students' ELBs, ELSE, and ECE with EALA and with one another. Although previous studies have demonstrated associations among learner beliefs, self-efficacy, positive emotions, and autonomous or self-regulated learning, these lines of research have often developed independently. In particular, relatively limited research has examined how cognitive beliefs, efficacy judgments, and positive classroom emotions are jointly associated with autonomous English learning, especially among adolescent learners.

The present study seeks to address this gap in three respects. First, it focuses on secondary school students, thereby extending a body of literature that has predominantly examined university students and adult EFL learners. Second, it integrates ELBs, ELSE, ECE, and EALA within a single moderated mediation model, distinguishing ELSE as a potential statistical mediator from ECE as an affective moderator. Third, by situating these relationships within the context of current curriculum reform and the Double Reduction Policy in China, the study links policy expectations regarding students' learning ability with the psychological factors associated with autonomous English learning.

**Figure 1:**
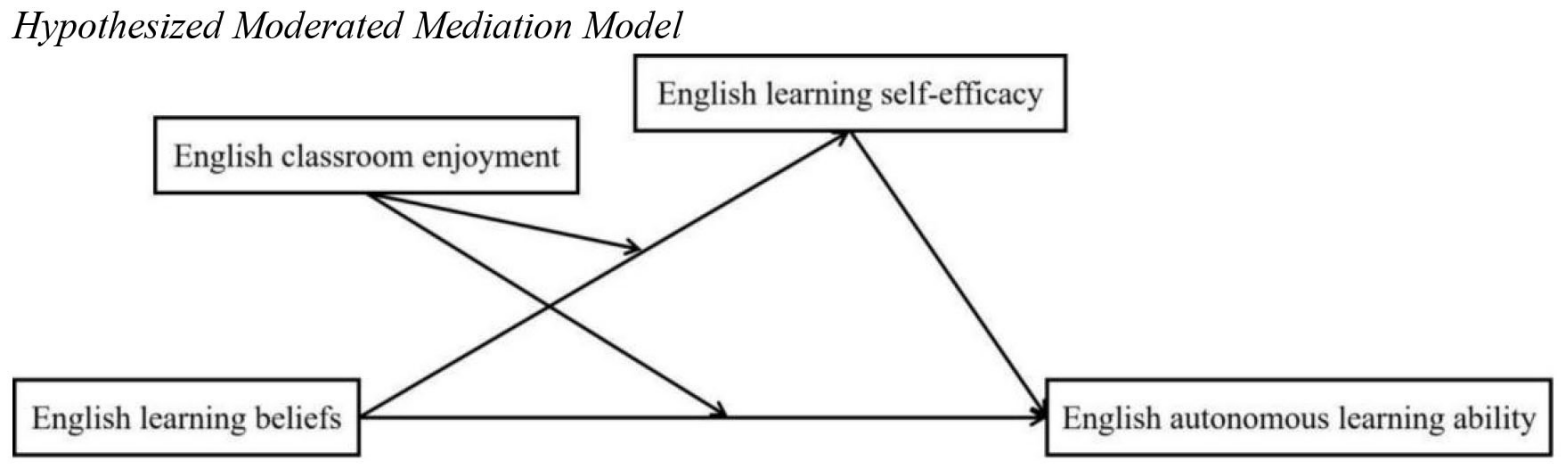
Hypothesized moderated mediation model.

Figure 1 presents the moderated mediation model based on the three hypotheses proposed above.

## Method

### Participants and procedure

A cross-sectional survey design was employed. Participants were recruited using convenience sampling from secondary schools across 11 provincial-level regions in China. After contact was established with participating schools, teachers assisted in distributing an online questionnaire to eligible students, who completed the survey independently after class. Participation was voluntary, and the questionnaire was completed anonymously. Before participating in the study, students were informed of the purpose and procedures of the study, the voluntary nature of participation, and their right to withdraw at any time without penalty. For participants under the age of 18, informed consent was obtained from their parents or legal guardians in addition to the students' assent. Participants aged 18 years or older provided informed consent themselves. The study was reviewed and approved by the Institutional Review Board of the Faculty of Psychology, Southwest University (Approval No. H21001).

A total of 2,217 students initially completed the questionnaire. Responses were screened for data quality before analysis. Questionnaires were excluded if the completion time was shorter than 300 s or longer than 2,000 s, or if systematic response patterns indicative of inattentive responding were identified, such as straight-lining or diagonal response patterns. No missing data were present in the submitted questionnaires due to the settings of the online survey platform. After data screening, 1,939 valid questionnaires were retained for the final analyses, corresponding to a valid response rate of 87.46%. Participants ranged in age from 10 to 20 years (*M* = 15.469, *SD* = 1.687). The sample included students from both junior and senior secondary schools; a cross-check of age and grade information showed that participants older than 18 years were enrolled in Grade 12. Considering possible variations in school-entry age and grade repetition, and because these participants otherwise met the eligibility and data-quality criteria, they were retained in the final sample. The socio-demographic characteristics of all participants are shown in Table 1.

**Table 1:** Sociodemographic characteristics of participants.

Characteristic	Frequency		Characteristic	Frequency	
	*n*	% Total		*n*	% Total
Provincial-level region			Gender		
Chongqing	873	45.02	Boys	903	46.57
Zhejiang Province	342	17.64	Girls	1,036	53.43
Inner Mongolia Autonomous Region	296	15.27	School stage		
			Junior secondary school	870	44.87
Sichuan Province	270	13.92	Senior secondary school	1069	55.13
Yunnan Province	126	6.50	Family residence		
Shanxi Province	15	0.77	Urban	1,338	69.00
Shanghai	11	0.57	Rural	601	31.00
Beijing	3	0.15	Monthly family income		
Anhui Province	1	0.05	Less than 1,000 RMB	36	1.86
Hubei Province	1	0.05	1,000–3,000 RMB	219	11.29
Taiwan	1	0.05	3,001–5,000 RMB	358	18.46
Only-child status			5,001–10,000 RMB	644	33.21
One-child family	709	36.57	10,001–20,000 RMB	446	23.00
Multiple-child family	1,230	63.43	More than 20,000 RMB	236	12.17

### Measures

All instruments were administered in Chinese. The present study used established Chinese-language measures that had either been originally developed in Chinese or previously adapted and validated for Chinese learners. No translation, back-translation, item addition, item deletion, or wording modification was conducted for any of the four measures in the present study. Because the established Chinese versions were used without modification, no separate pilot test was conducted before the main survey. All measures retained their original item content and response formats.

For all four constructs, an overall mean score across all items was calculated and used in the subsequent statistical analyses. Reverse-keyed items, where applicable, were reverse-scored before the overall mean score was calculated. Thus, the composite scores for all four constructs remained on the original 1–5 metric.

#### English learning beliefs scale

ELBs were assessed using the Chinese version of Horwitz's Beliefs About Language Learning Inventory (BALLI; Horwitz, 1985) adapted by Chen (2008). This Chinese version has subsequently been used with Chinese secondary-school students (Duan, 2019). The scale consists of 32 items covering five dimensions: foreign language aptitude, difficulty of English learning, the nature of English learning, learning and communication strategies, and motivation and expectations. Participants rated each item on a five-point Likert scale ranging from 1 (“strongly disagree”) to 5 (“strongly agree”). The scale contains no reverse-keyed items. Following the scoring approach used in previous studies, an overall mean score across the 32 items was calculated to represent participants' overall ELBs. In the present study, the scale demonstrated good internal consistency (Cronbach's α = 0.905). A confirmatory factor analysis was conducted to examine the factorial structure of the ELBs scale. The model showed an overall acceptable fit to the data (CFI = 0.917, TLI = 0.898, RMSEA = 0.059, 90% CI [0.057, 0.061], SRMR = 0.052).

#### English learning self-efficacy scale

ELSE was assessed using the Chinese English Learning Self-Efficacy Scale developed by Zhang and Yuan (2004). The scale was originally developed in Chinese and has subsequently been used in research involving Chinese middle-school students (Peng, 2021). The scale consists of 22 items covering four dimensions: ability to complete English learning tasks, confidence in achieving English learning goals, ability to cope with setbacks in English learning, and ability to overcome English learning difficulties. Participants rated each item on a five-point Likert scale ranging from 1 (“strongly disagree”) to 5 (“strongly agree”). Items 3, 6, 9, 12, 17, and 21 are reverse-keyed. These items were reverse-scored before the scale score was calculated (i.e., 1 = 5, 2 = 4, 3 = 3, 4 = 2, and 5 = 1). After reverse scoring, an overall mean score across all 22 items was calculated to represent participants' overall ELSE. In the present study, the scale demonstrated good internal consistency (Cronbach's α = 0.928). A confirmatory factor analysis was conducted to examine the factorial structure of the ELSE scale. The model showed an overall acceptable fit to the data (CFI = 0.940, TLI = 0.929, RMSEA = 0.067, 90% CI [0.065, 0.070], SRMR = 0.049).

#### English autonomous learning ability questionnaire

EALA was assessed using the Chinese English Autonomous Learning Ability Questionnaire developed for Chinese secondary-school students (Liu, 2020). The questionnaire consists of 33 items covering five dimensions: learning motivation, learning time, learning strategies, learning process, and learning outcomes and environment. Participants rated each item on a five-point Likert scale ranging from 1 (“strongly disagree”) to 5 (“strongly agree”). The questionnaire contains no reverse-keyed items. An overall mean score across all 33 items was calculated to represent participants' overall EALA. In the present study, the questionnaire demonstrated good internal consistency (Cronbach's α = 0.975). A confirmatory factor analysis was conducted to examine the factorial structure of the EALA scale. The model showed an overall acceptable fit to the data (CFI = 0.900, TLI = 0.890, RMSEA = 0.075, 90% CI [0.073, 0.077], SRMR = 0.047).

#### English classroom enjoyment scale

ECE was assessed using the established Chinese version of the Foreign Language Classroom Enjoyment Scale developed and validated by Jin and Zhang (2019). The Chinese version has also been used in research involving Chinese secondary-school students (Cheng, 2019). The scale consists of 16 items. Participants rated each item on a five-point Likert scale ranging from 1 (“strongly disagree”) to 5 (“strongly agree”), with 3 representing “neither agree nor disagree.” The scale contains no reverse-keyed items. An overall mean score across all 16 items was calculated to represent participants' overall ECE. In the present study, the scale demonstrated good internal consistency (Cronbach's α = 0.945). A confirmatory factor analysis was conducted to examine the factorial structure of the ECE scale. The model showed an overall acceptable fit to the data (CFI = 0.954, TLI = 0.943, RMSEA = 0.077, 90% CI [0.073, 0.081], SRMR = 0.039).

### Data analysis

Data were analyzed using SPSS 26.0. Hayes' PROCESS macro (Version 3.3) was used to examine the statistical mediation (Model 4) and moderated mediation (Model 8) models. All continuous variables (ELBs, ELSE, ECE, EALA, and age) were standardized as Z scores (M = 0, SD = 1) before the regression analyses. School stage, family residence, and only-child status were entered as dichotomous variables, whereas monthly family income was treated as an ordinal variable. Bootstrap procedures with 5,000 resamples and 95% percentile bootstrap confidence intervals were used to evaluate the indirect association and the index of moderated mediation. An estimate was considered statistically significant when its corresponding 95% confidence interval did not include zero.

Before testing the hypothesized mediation and moderated mediation models, the assumptions underlying the regression analyses were examined, including multicollinearity, normality, homoscedasticity, and influential observations. Multicollinearity was assessed using tolerance and variance inflation factor (VIF) statistics. Normality was evaluated using the skewness and kurtosis of the standardized residuals, together with standardized residual histograms and normal P-P plots. Homoscedasticity was assessed by inspecting plots of standardized residuals against standardized predicted values, and potentially influential observations were examined using Cook's distance.

## Results

### Preliminary analyses

Because all variables in the present study were assessed using self-report measures, Harman's single-factor test was conducted to examine potential common method bias. The results showed that 12 factors had eigenvalues greater than 1, and the first factor accounted for 37.50% of the total variance, which was below the 40% threshold, suggesting that substantial common method variance was not indicated by this test. In addition, across the regression equations, tolerance values ranged from 0.288 to 0.983, and VIF values ranged from 1.018 to 3.471, indicating no serious multicollinearity.

The skewness values of the standardized residuals ranged from −0.563 to −0.242, and the kurtosis values ranged from 2.160 to 3.749. Together with inspection of the standardized residual histograms and normal P-P plots, these results indicated no substantial deviation from normality. Furthermore, scatterplots of standardized residuals against standardized predicted values revealed no significant violation of homoscedasticity. Finally, the maximum Cook's distance values ranged from 0.099 to 0.117, indicating that no individual observation exerted an undue influence on the regression estimates.

### Correlational analysis and tests for differences

As shown in Table 2, ELBs, ELSE, ECE, and EALA were significantly and positively correlated with one another (all *p* < 0.001), providing preliminary support for the hypothesized relationships examined in the subsequent analyses. Age was negatively correlated with ELSE, ECE, and EALA, but was not significantly correlated with ELBs. In addition, the tests for differences presented in Table 3 showed significant differences in ELSE and/or EALA across school stage, only-child status, family residence, and monthly family income. Based on these results and the demographic characteristics considered in the study, age, school stage, only-child status, family residence, and monthly family income were included as covariates in the subsequent statistical mediation and moderated mediation analyses.

**Table 2:** Descriptive statistics and correlations among the study variables.

Variable	M	SD	1	2	3	4	5
1. ELBs	3.513	0.483	1				
2. ECE	3.756	0.693	0.631^***^	1			
3. ELSE	3.342	0.607	0.668^***^	0.742^***^	1		
4. EALA	3.494	0.689	0.741^***^	0.756^***^	0.820^***^	1	
5. Age	15.469	1.687	−0.033	−0.095^***^	−0.078^***^	−0.126^***^	1

ELBs, English learning beliefs; ECE, English classroom enjoyment; ELSE, English learning self-efficacy; EALA, English autonomous learning ability.

^***^p < 0.001.

**Table 3:** Differences in ELSE and EALA scores by sociodemographic characteristics.

Variable	ELSE		EALA	
	*M*	*SD*	*M*	*SD*
Gender				
Boys	3.345	0.619	3.469	0.737
Girls	3.339	0.596	3.515	0.644
*t*	0.239		−1.469	
Only-child status				
One-child family	3.435	0.608	3.570	0.683
Multiple-child family	3.289	0.599	3.450	0.698
*t*	−5.123^***^		−3.706^***^	
School stage				
Junior secondary school	3.375	0.633	3.576	0.733
Senior secondary school	3.315	0.582	3.427	0.644
*t*	2.134^*^		4.713^***^	
Family residence				
Rural	3.195	0.570	3.366	0.695
Urban	3.409	0.611	3.552	0.679
*t*	−7.460^***^		−5.543^***^	
Monthly family income				
Less than 1,000 RMB	3.061	0.651	3.262	0.752
1,000–3,000 RMB	3.152	0.528	3.344	0.644
3,001–5,000 RMB	3.188	0.552	3.382	0.655
5,001–10,000 RMB	3.353	0.608	3.498	0.682
10,000–20,000 RMB	3.467	0.607	3.589	0.672
More than 20,000 RMB	3.533	0.623	3.649	0.760
*F*	19.985^***^		9.071^***^	

ELSE, English learning self-efficacy; EALA, English autonomous learning ability.

^*^p < 0.05,

^***^p < 0.001.

For the regression analyses, school stage, only-child status, and family residence were treated as categorical variables and dummy-coded. Monthly family income was treated as an ordinal covariate and coded from 1 to 6 according to the six ordered income categories, with higher values representing higher levels of family income. This coding was used to capture the ordered trend across income categories (Williams, 2020). Although age and school stage are related, both were retained because they capture conceptually distinct characteristics: age reflects developmental and maturational differences among students, whereas school stage represents their educational stage and corresponding instructional experience (Cahan and Cohen, 1989; Morrison et al., 2019).

### Simple mediation analysis

PROCESS Model 4 was used to test the statistical mediation of ELSE in the association between ELBs and EALA, with ELBs entered as the independent variable (X), ELSE as the mediator (M), and EALA as the dependent variable (Y). School stage, age, family residence, only-child status, and monthly family income were included as covariates in the analysis.

As shown in Table 4, ELBs were positively associated with EALA (*B* = 0.731, *SE* = 0.015, *t* = 48.103, *p* < 0.001, 95% CI [0.701, 0.761]). After ELSE and the covariates were entered into the model, the direct association between ELBs and EALA remained significant but was smaller (*B* = 0.354, *SE* = 0.015, *t* = 22.979, *p* < 0.001, 95% CI [0.324, 0.384]). ELBs were positively associated with ELSE (*B* = 0.647, *SE* = 0.017, *t* = 38.390, *p* < 0.001, 95% CI [0.614, 0.680]), and ELSE was positively associated with EALA (*B* = 0.583, *SE* = 0.016, *t* = 37.249, *p* < 0.001, 95% CI [0.552, 0.613]).

**Table 4:** Statistical mediation analysis of ELSE (*N* = 1,939).

Outcome	Predictor	*B^*a*^*	*SE*	*t*	*p*	95% CI		*R* ^2^	*F*
						LLCI	ULCI		
EALA	ELBs	0.731	0.015	48.103	< 0.001	0.701	0.761	0.565	418.675^***^
(Model 1)	Age	−0.033	0.028	−1.183	0.237	−0.088	0.022		
	School stage^b^	−0.160	0.056	−2.845	0.004	−0.270	−0.050		
	Family residence^b^	0.065	0.036	1.830	0.067	−0.005	0.135		
	Only-child status^b^	0.072	0.032	2.243	0.025	0.009	0.135		
	Monthly family income^b^	0.022	0.013	1.623	0.105	−0.005	0.048		
ELSE	ELBs	0.647	0.017	38.390	< 0.001	0.614	0.680	0.465	279.479^***^
(Model 2)	Age	−0.040	0.031	−1.291	0.197	−0.100	0.021		
	School stage^b^	−0.017	0.062	−0.270	0.787	−0.139	0.105		
	Family residence^b^	0.073	0.040	1.839	0.066	−0.005	0.151		
	Only-child status^b^	0.091	0.036	2.562	0.011	0.021	0.161		
	Monthly family income^b^	0.076	0.015	5.168	< 0.001	0.047	0.105		
EALA	ELBs	0.354	0.015	22.979	< 0.001	0.324	0.384	0.747	814.623^***^
(Model 3)	ELSE	0.583	0.016	37.249	< 0.001	0.552	0.613		
	Age	−0.010	0.021	−0.457	0.648	−0.051	0.032		
	School stage^b^	−0.150	0.043	−3.500	< 0.001	−0.234	−0.066		
	Family residence^b^	0.023	0.027	0.839	0.402	−0.031	0.076		
	Only-child status^b^	0.019	0.025	0.767	0.443	−0.029	0.067		
	Monthly family income^b^	−0.023	0.010	−2.236	0.025	−0.043	−0.003		

ELBs, English learning beliefs; ECE, English classroom enjoyment; ELSE, English learning self-efficacy; EALA, English autonomous learning ability.

^a^B represents the unstandardized regression coefficient estimated by PROCESS. ELBs, ELSE, EALA, and age were z-standardized before analysis; therefore, coefficients involving these continuous variables were estimated on their standardized metrics.

^b^School stage was coded as 0 = junior secondary school and 1 = senior secondary school (reference = junior secondary school). Family residence was coded as 0 = rural and 1 = urban (reference = rural). Only-child status was coded as 0 = one-child family and 1 = multiple-child family (reference = one-child family). Monthly family income was treated as an ordinal variable coded from 1 to 6.

^***^p < 0.001.

As shown in Table 5, the direct association between ELBs and EALA was significant (*B* = 0.354, *SE* = 0.015, 95% CI [0.324, 0.384]). The indirect association through ELSE was also significant (indirect association = 0.377, BootSE = 0.019, 95% bootstrap CI [0.340, 0.414]), as the bootstrap confidence interval did not include zero. These results indicated that ELBs were associated with EALA both directly and indirectly through ELSE. The direct and indirect components accounted for 48.427% and 51.573% of the total association (0.731), respectively.

**Table 5:** Decomposition of the total, direct, and indirect associations.

Type	Path	Effect	SE	95% CI		% of total association
				Lower	Upper	
Total association	ELBs → EALA	0.731	0.015	0.701	0.761	
Direct association	ELBs → EALA	0.354	0.015	0.324	0.384	48.427
Indirect association through ELSE	ELBs → ELSE → EALA	0.377	0.019	0.340	0.414	51.573

ELBs, English learning beliefs; ELSE, English learning self-efficacy; EALA, English autonomous learning ability.

BootSE and percentile bootstrap 95% confidence intervals are reported for the indirect association.

### Moderated mediation analysis

PROCESS Model 8 was used to test the moderated mediation model (Table 6), with ELBs entered as the independent variable (X), ELSE as the mediator (M), ECE as the moderator (W), and EALA as the dependent variable (Y). School stage, age, family residence, only-child status, and monthly family income were included as covariates in the analysis.

**Table 6:** Moderated mediation analysis.

Outcome	Predictor	*B^*a*^*	*SE*	*t*	*p*	95% CI		*R* ^2^	*F*
						LLCI	ULCI		
ELSE	ELBs	0.333	0.018	18.487	< 0.001	0.298	0.369	0.626	403.580^***^
	ECE	0.520	0.018	28.641	< 0.001	0.485	0.556		
	ELBs × ECE	0.036	0.010	3.761	< 0.001	0.017	0.054		
	Age	−0.006	0.026	−0.243	0.808	−0.057	0.044		
	School stage^b^	−0.003	0.052	−0.060	0.952	−0.105	0.099		
	Family residence^b^	0.021	0.033	0.643	0.521	−0.044	0.087		
	Only-child status^b^	0.055	0.030	1.845	0.065	−0.004	0.114		
	Monthly family income^b^	0.053	0.012	4.269	< 0.001	0.029	0.077		
EALA	ELBs	0.298	0.015	19.466	< 0.001	0.268	0.329	0.770	718.595^***^
	ELSE	0.446	0.018	25.002	< 0.001	0.411	0.481		
	ECE	0.237	0.017	13.943	< 0.001	0.204	0.270		
	ELBs × ECE	0.012	0.008	1.588	0.113	−0.003	0.027		
	Age	0.000	0.020	0.010	0.992	−0.040	0.040		
	School stage^b^	−0.147	0.041	−3.595	< 0.001	−0.227	−0.067		
	Family residence^b^	0.009	0.026	0.346	0.730	−0.042	0.060		
	Only-child status^b^	0.015	0.023	0.627	0.531	−0.031	0.061		
	Monthly family income^b^	−0.023	0.010	−2.347	0.019	−0.042	−0.004		

ELBs, English learning beliefs; ECE, English classroom enjoyment; ELSE, English learning self-efficacy; EALA, English autonomous learning ability.

^a^B represents the unstandardized regression coefficient estimated by PROCESS. ELBs, ECE, ELSE, EALA, and age were z-standardized before analysis; therefore, coefficients involving these continuous variables were estimated on their standardized metrics.

^b^School stage was coded as 0 = junior secondary school and 1 = senior secondary school (reference = junior secondary school). Family residence was coded as 0 = rural and 1 = urban (reference = rural). Only-child status was coded as 0 = one-child family and 1 = multiple-child family (reference = one-child family). Monthly family income was treated as an ordinal variable coded from 1 to 6.

^***^p < 0.001.

The interaction between ELBs and ECE was significantly associated with ELSE (*B* = 0.036, *SE* = 0.010, *t* = 3.761, *p* < 0.001, 95% CI [0.017, 0.054]), indicating that ECE significantly moderated the association between ELBs and ELSE. In contrast, the interaction between ELBs and ECE was not significantly associated with EALA (*B* = 0.012, *SE* = 0.008, *t* = 1.588, *p* = 0.113, 95% CI [−0.003, 0.027]), indicating that ECE did not significantly moderate the direct association between ELBs and EALA.

**Figure 2:**
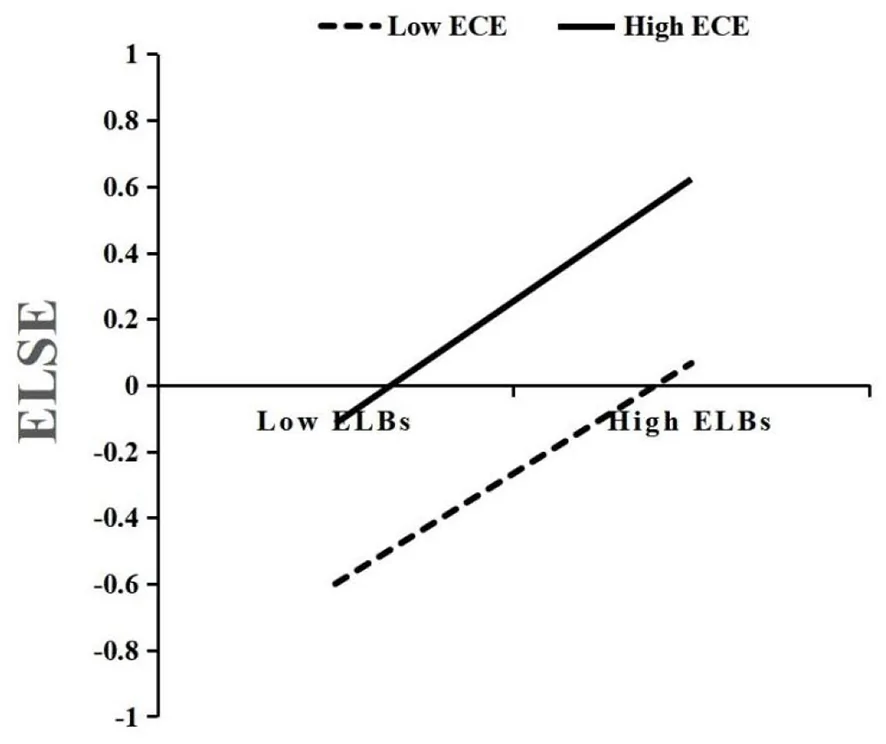
Conditional association between ELBs and ELSE at different levels of ECE. ELBs, English learning beliefs; ECE, English classroom enjoyment; ELSE, English learning self-efficacy.

To further examine the interaction between ELBs and ECE, the conditional associations between ELBs and ELSE were estimated at one standard deviation below the mean, at the mean, and one standard deviation above the mean of ECE. A simple slopes analysis showed that at lower levels of ECE (1 *SD* below the mean), the positive association between ELBs and ELSE was weaker (*B* = 0.298, *SE* = 0.020, *t* = 15.064, *p* < 0.001, 95% CI [0.259, 0.336]), whereas at higher levels of ECE (1 *SD* above the mean), the association was stronger (*B* = 0.369, *SE* = 0.021, *t* = 17.596, *p* < 0.001, 95% CI [0.328, 0.410]; Figure 2).

The conditional indirect associations of ELBs with EALA through ELSE were significant at low (indirect association = 0.133, BootSE = 0.016, 95% bootstrap CI [0.104, 0.166]), mean (indirect association = 0.149, BootSE = 0.015, 95% bootstrap CI [0.121, 0.179]), and high levels of ECE (indirect association = 0.165, BootSE = 0.015, 95% bootstrap CI [0.136, 0.195]; Table 7). Moreover, the index of moderated mediation was significant (index = 0.016, BootSE = 0.004, 95% bootstrap CI [0.007, 0.024]), indicating that the indirect association between ELBs and EALA through ELSE varied significantly as a function of ECE.

**Table 7:** Conditional indirect association through ELSE at different levels of ECE.

Moderator levels	Type	Effect	Estimate	SE/BootSE	95% CI	
ECE					Lower	Upper
M – 1 SD	Component	ELBs → ELSE	0.298	0.020	0.259	0.336
M	Component	ELBs → ELSE	0.333	0.018	0.298	0.369
M + 1 SD	Component	ELBs → ELSE	0.369	0.021	0.328	0.410
M – 1 SD	Indirect association	ELBs → ELSE → EALA	0.133	0.016	0.104	0.166
M	Indirect association	ELBs → ELSE → EALA	0.149	0.015	0.121	0.179
M + 1 SD	Indirect association	ELBs → ELSE → EALA	0.165	0.015	0.136	0.195
Index of moderated mediation			0.016	0.004	0.007	0.024

ELBs, English learning beliefs; ECE, English classroom enjoyment; ELSE, English learning self-efficacy; EALA, English autonomous learning ability. For the component effects, Estimate represents the unstandardized regression coefficient (B), and SE represents the standard error. For the indirect effects and the index of moderated mediation, BootSE and percentile bootstrap 95% confidence intervals based on 5,000 bootstrap samples are reported.

Thus, Hypothesis 1 and Hypothesis 2 were supported, whereas Hypothesis 3 was partially supported: ECE moderated the association between ELBs and ELSE but not the direct association between ELBs and EALA.

## Discussion

Based on survey data from secondary school students in 11 provincial-level regions of China, including Chongqing, Zhejiang, and Sichuan, the present study found that ELBs were positively associated with EALA, that there was a significant indirect association between ELBs and EALA through ELSE, and that ECE moderated the association between ELBs and ELSE. These results extend the existing literature on English autonomous learning and classroom enjoyment and provide insights into the psychological factors associated with students' EALA.

### Secondary school students' ELBs and EALA

The results showed that secondary school students' ELBs were positively associated with their EALA, supporting Hypothesis 1.

In psychological research, beliefs are usually considered higher-order cognitions that are closely associated with individuals' attitudes and behaviors. Self-regulation theory posits that an individual's beliefs about knowledge and learning are closely involved in how they approach and regulate learning (Zimmerman, 2000). ELBs reflect how learners interpret the nature of language ability, the difficulty of English learning, the usefulness of learning and communication strategies, and the value and purposes of learning. These interpretations may provide cognitive reference points for decisions that are central to autonomous learning. For example, students who regard language ability as malleable rather than fixed may be more willing to persist when difficulties arise and to interpret temporary failure as part of the learning process. Similarly, students who recognize that successful language learning requires active communication and strategic practice may be more likely to plan their learning, select appropriate strategies, seek additional learning resources, and evaluate the effectiveness of their approaches. By contrast, beliefs that language ability is largely innate or that successful learning depends primarily on external instruction may be accompanied by greater dependence on teachers and fewer self-regulatory behaviors. Thus, the positive association observed in the present study may be understood as reflecting the relevance of ELBs as cognitive frameworks for how students interpret learning demands and regulate their own learning.

These findings and interpretations are broadly consistent with international research. For instance, prior research has identified a significant association between high school students' epistemic beliefs and their learning strategies (Shirzad et al., 2022). Furthermore, learners' beliefs are associated with how they interpret and engage with emerging learning opportunities, including informal English learning in AI-mediated contexts (Wang et al., 2025). The results of the present study further suggest that learning beliefs are associated not only with the use of specific learning strategies but also with EALA as a broader learning capacity.

### Moderated mediation

#### The mediating role of ELSE

The results showed a significant indirect association between ELBs and EALA through ELSE, while the direct association between ELBs and EALA remained significant. Thus, Hypothesis 2 was supported.

Similarly, prior research has reported an indirect association between epistemic beliefs and language-learning strategies through self-efficacy (Shirzad et al., 2022), while self-efficacy has also been found to be significantly associated with students' autonomous technology use in second language learning (Csizér and Albert, 2024). From the perspectives of social cognitive theory and self-regulated learning, self-efficacy is closely related to effort, persistence, strategy selection, and learning behaviors (Bandura, 1993, 1997; Zimmerman, 2000). Therefore, one plausible interpretation is that more adaptive ELBs may be associated with a more favorable cognitive basis for students' evaluations of their own learning capabilities. Students who believe that English proficiency can develop through effort and effective strategy use may be more inclined to view learning difficulties as surmountable challenges rather than evidence of inadequate ability. Such cognitive appraisals may be associated with stronger self-efficacy judgments. Higher ELSE is also associated with a greater tendency to set goals, persist in the face of setbacks, experiment with diverse strategies, and independently monitor learning processes (Mo, 2020)—behaviors that closely align with the core features of EALA.

#### The moderating role of ECE

The results showed that ECE significantly moderated the association between ELBs and ELSE. However, ECE did not significantly moderate the direct association between ELBs and EALA. Thus, Hypothesis 3 was only partially supported.

This finding also appears to be broadly consistent with Control-Value Theory (Pekrun, 2006) and the Broaden-and-Build Theory (Fredrickson, 2001). As a positive academic emotion, ECE is closely related to the classroom environment (Dewaele and MacIntyre, 2016; Jin and Zhang, 2021). According to Control-Value Theory, positive task-related emotions are theorized to be particularly relevant to proximal appraisal processes, such as perceived control and self-efficacy. At the same time, the Broaden-and-Build Theory proposes that positive emotional experiences can broaden learners' attention and facilitate the use of existing cognitive and psychological resources (Fredrickson, 2000, 2001). From this perspective, more adaptive and positive ELBs may represent useful cognitive resources, while ECE may be viewed as an affective condition associated with variation in the strength of the relationship between these beliefs and positive self-evaluations. When students enjoy their English classes, they may be more willing to participate, attempt challenging tasks, interpret classroom experiences more positively, and recognize evidence of their own competence. Accordingly, the stronger association between ELBs and ELSE at higher levels of ECE may be consistent with a classroom context in which adaptive learning beliefs are more strongly associated with students' evaluations of their own learning capabilities (Li et al., 2020).

On the other hand, the nonsignificant moderating role of ECE in the direct association between ELBs and EALA also warrants particular attention. ELSE represents a relatively proximal self-evaluation of one's capability to accomplish English learning tasks and may therefore be especially sensitive to students' current emotional experiences in the classroom. In contrast, EALA reflects a more complex and enduring pattern of learning behavior that extends beyond the immediate classroom context (Zimmerman, 2000; Teng and Zhang, 2022). As a context-sensitive emotional state, ECE may therefore be less strongly associated with variation in the strength of the relationship between ELBs and EALA.

This finding extends existing research on language learning (e.g., Li and Wei, 2023; Villavicencio and Bernardo, 2016) by positioning enjoyment as an affective boundary condition rather than a factor uniformly associated with autonomous learning. Cognitive beliefs, efficacy judgments, and classroom emotions may therefore show distinct yet complementary associations with students' autonomous English learning.

### Limitations and future research opportunities

Although the present study contributes to research on EALA among secondary school students, several limitations should be acknowledged. First, although the participants were recruited from secondary schools across 11 provincial-level regions in China, convenience sampling was employed. Therefore, the sample may be subject to some degree of selection bias and may not fully represent the broader population of Chinese secondary school students. Accordingly, caution is warranted when generalizing the present findings. Future research could employ stratified or multistage probability sampling and include students from a wider range of geographic regions, school types, and socioeconomic backgrounds to improve sample representativeness.

Second, the present study adopted a cross-sectional survey design. Although the analyses revealed significant associations among ELBs, ELSE, ECE, and EALA, the temporal ordering and causal relationships among these variables cannot be established. Accordingly, the indirect association through ELSE should be interpreted as statistical mediation rather than evidence of a causal mechanism. Reverse or reciprocal relationships among the variables also remain possible. Future research could employ longitudinal or cross-lagged designs to examine the temporal ordering and reciprocal relationships among these variables. Experimental or intervention studies could further determine whether changes in learning beliefs, self-efficacy, or classroom enjoyment lead to subsequent changes in autonomous learning.

Third, all study data were collected through student self-report questionnaires administered at a single time point. Although procedural controls and statistical diagnostics did not indicate substantial common method bias, self-report measures remain susceptible to factors such as social desirability bias and recall bias. Moreover, common method effects cannot be completely ruled out because all focal variables were obtained from the same respondents at the same measurement occasion. Future research could adopt multi-source and multi-method approaches by combining student self-reports with teacher or parent assessments, classroom observations, learning logs, or behavioral indicators of autonomous learning. Collecting different variables at different time points may also help reduce potential common method bias.

Fourth, the study was conducted within the specific sociocultural and educational context of China, where English is primarily learned as a foreign language and where ongoing curriculum reform and the Double Reduction Policy form part of the educational context in which students' learning takes place. The associations among learning beliefs, self-efficacy, classroom enjoyment, and autonomous learning may vary across educational systems, cultural contexts, and language-learning environments. Therefore, the present findings should not be directly generalized to learners in other countries, age groups, or instructional contexts. Cross-cultural and cross-national research is needed to examine whether the relationships identified in this study remain consistent across different educational and cultural settings.

Fifth, the clustered structure of the data could not be explicitly modeled. Participants were recruited through schools, and students were therefore potentially nested within schools and classes. However, school names were self-reported by students and included numerous abbreviations, incomplete entries, and spelling inconsistencies, which prevented reliable identification and matching of schools. In addition, class-level identifiers were not collected. Consequently, the available data did not permit the construction of reliable school- or class-level clustering variables, and multilevel modeling or cluster-robust standard errors could not be applied. If observations within the same school or class were correlated, the assumption of independent observations may have been partially violated, which could have affected the estimated standard errors and statistical significance of the results. Future research should collect standardized school and class identifiers at the time of data collection and employ multilevel models or cluster-robust estimation procedures to account explicitly for the hierarchical structure of students nested within classes and schools.

## Implications

The findings have several practical implications for secondary school English teaching. Given the cross-sectional nature of the study, these recommendations should be understood as pedagogical directions consistent with the observed associations rather than as interventions whose causal effectiveness has been established.

First, the positive association between ELBs and EALA suggests that teachers may consider paying greater attention to students' beliefs about how English is learned. Brief belief questionnaires, reflective prompts, or small-group discussions may be used to identify potentially maladaptive beliefs, such as viewing language ability as fixed, interpreting mistakes as evidence of low ability, or relying excessively on memorization and translation. Teachers might also incorporate opportunities for belief reflection into regular classroom activities. For example, after a listening, speaking, reading, or writing task, students can be asked to identify the strategies they used, evaluate their effectiveness, and reflect on how effort and strategy adjustment were related to their task performance. Learning journals or short strategy-reflection sheets may provide opportunities for students to consider the relationships among learning outcomes, controllable factors, and responsibility for their learning.

Second, the significant indirect association between ELBs and EALA through ELSE suggests that students' confidence in completing English-learning tasks deserves attention in instructional practice. Teachers may consider structuring tasks to provide opportunities for successful performance, for example, by breaking complex tasks into manageable steps and setting proximal, moderately challenging goals. For instance, speaking activities can progress from individual preparation to pair practice and then whole-class presentation. Strategy demonstrations, peer models with comparable proficiency, and process-focused feedback may also provide students with information about the potential importance of effective strategy use and sustained effort for learning progress rather than viewing ability as fixed. Progress records, weekly goals, portfolios, and brief self-evaluation checklists may provide additional opportunities for students to recognize their own learning progress and evaluate their capability to manage English-learning tasks.

Third, the moderating role of ECE suggests that classroom enjoyment may be considered a facilitating emotional condition rather than an instructional end in itself. Because ECE was associated with a stronger relationship between ELBs and ELSE but did not significantly moderate the direct association between ELBs and EALA, simply making lessons “fun” may be insufficient as a basis for addressing autonomous learning in instruction. Instead, teachers may consider combining enjoyable classroom experiences with meaningful participation and manageable challenge. Pair and group communication, information-gap activities, role plays, collaborative problem-solving, and project-based tasks can provide opportunities for active language use. Teachers may also seek to create an error-tolerant climate in which mistakes are treated as a normal part of learning. For students who are reluctant to participate publicly, pair or small-group rehearsal before whole-class performance may offer a lower-pressure context and additional opportunities for participation.

Overall, secondary school English instruction may benefit from integrating cognitive, motivational, and emotional support. Teachers may attend to students' learning beliefs, provide structured learning experiences relevant to self-efficacy, and foster supportive and enjoyable classroom environments. Such coordinated practices are consistent with conditions that may be relevant to greater autonomy in English learning.

## Conclusions

Our cross-sectional survey study investigated the associations among ELBs, ELSE, ECE, and EALA among secondary school students. The results revealed that ELBs were significantly and positively associated with EALA, with a significant indirect association between ELBs and EALA through ELSE. ECE also significantly moderated the association between ELBs and ELSE, whereas its moderating role in the direct association between ELBs and EALA was not significant. These results supported Hypotheses 1 and 2 and partially supported Hypothesis 3.

## Data Availability

The raw data supporting the conclusions of this article will be made available by the authors, without undue reservation.
